# Episodic Memory and Appetite Regulation in Humans

**DOI:** 10.1371/journal.pone.0050707

**Published:** 2012-12-05

**Authors:** Jeffrey M. Brunstrom, Jeremy F. Burn, Nicola R. Sell, Jane M. Collingwood, Peter J. Rogers, Laura L. Wilkinson, Elanor C. Hinton, Olivia M. Maynard, Danielle Ferriday

**Affiliations:** Nutrition and Behaviour Unit, School of Experimental Psychology, University of Bristol, Bristol, United Kingdom; Pennington Biomedical Research Center, United States of America

## Abstract

Psychological and neurobiological evidence implicates hippocampal-dependent memory processes in the control of hunger and food intake. In humans, these have been revealed in the hyperphagia that is associated with amnesia. However, it remains unclear whether ‘memory for recent eating’ plays a significant role in neurologically intact humans. In this study we isolated the extent to which memory for a recently consumed meal influences hunger and fullness over a three-hour period. Before lunch, half of our volunteers were shown 300 ml of soup and half were shown 500 ml. Orthogonal to this, half consumed 300 ml and half consumed 500 ml. This process yielded four separate groups (25 volunteers in each). Independent manipulation of the ‘actual’ and ‘perceived’ soup portion was achieved using a computer-controlled peristaltic pump. This was designed to either refill or draw soup from a soup bowl in a covert manner. Immediately after lunch, self-reported hunger was influenced by the actual and not the perceived amount of soup consumed. However, two and three hours after meal termination this pattern was reversed - hunger was predicted by the perceived amount and not the actual amount. Participants who thought they had consumed the larger 500-ml portion reported significantly less hunger. This was also associated with an increase in the ‘expected satiation’ of the soup 24-hours later. For the first time, this manipulation exposes the independent and important contribution of memory processes to satiety. Opportunities exist to capitalise on this finding to reduce energy intake in humans.

## Introduction

Obesity remains a major health concern [1]. Therefore, understanding controls of energy intake should be given a high priority. The prevailing view is that meal size is governed by homeostatic neural and endocrine signals that are detected in the hypothalamus and hindbrain [2]. However, it is increasingly recognised that energy balance is also influenced by higher neural systems [3]. In particular, the hippocampus has attracted attention because it receives input from the hypothalamus and because leptin and insulin receptors are expressed in this region [4], [5]. The hippocampus plays an important role in learning and memory [6] and hippocampal lesioned rats display increased ‘interoceptive agnosia’ [7]. Together, these observations provide evidence that hippocampal-dependent memory mechanisms help to mobilise behavioural responses to food [8]–[12].

The prospect that memory plays an important role in the regulation of food intake is consistent with an emerging literature on ‘memory for recent eating’ in humans [13], [14]. In a series of studies, Higgs and colleagues have shown that reminding people of a recent meal can decrease the amount that is consumed at a subsequent meal [13], [15]. This effect persists for several hours into the inter-meal interval and, importantly, it is evident only when memory of a very recent meal is recalled.

With this paradigm, a potential concern is that the effect on food intake is revealed only after an instruction to recall a recent meal. Whether humans routinely retrieve explicit memories of recent meals remains unclear. An alternative strategy is to disrupt memory encoding during a meal and then measure appetite and intake at a subsequent meal. This can be achieved by asking volunteers to engage in a distracting task (*e.g.*, watching television or playing a computer game) while eating. This not only impairs the quality of memory for the meal but it is associated with elevated hunger in the inter-meal interval and with greater consumption at a subsequent meal [16], [17]. Again, a difficulty with this approach is that the effect of distraction on memory formation is very difficult to measure or confirm with certainty. Moreover, distraction has the potential to influence eating rate, mood, and level of stress, all of which are known to moderate appetite and food intake [18]–[20].

Perhaps the most striking evidence for impaired encoding is found in patients with retrograde amnesia. Again, consistent with Higgs’s interpretation, bilateral hippocampal damage is associated with hyperphagia - after eating one meal to fullness, an amnesic may go on to consume further meals and to report no memory for recent eating and little change in hunger [21]–[23]. Albeit dramatic, to date, these behaviours have only been documented in a small number of individuals, many of whom had structural deficits that extended beyond the hippocampus (amygdala and insula). More generally, neuroscientists widely acknowledge the limitations of inferring normal cognitive function from examples of neurological impairment [24].

Together, these findings highlight a potentially important role for episodic memory in the control of meal size and appetite in humans. This merits attention, not least because it challenges our understanding of short-term energy regulation and, in particular, the long-standing assumption that cognition plays a role primarily around a meal and during the early stages of the ‘satiety cascade’ [25]. However, to test this hypothesis directly, a novel paradigm is required. Specifically, one that can both isolate and quantify the effects of episodic memory on satiety, relative to other psychobiological influences. For the first time, we present an empirical approach that creates this dissociation, under relatively normal eating conditions, and in neurologically intact humans.

Previously, Wansink *et al.* have used a passive self-refilling soup bowl to remove visual information about the amount of soup that is consumed in a meal [26]. In the present study we used an adapted version of this paradigm. Specifically, we developed a process that enables the experimenter to either increase or decrease a predetermined amount of soup into a soup bowl. This was achieved covertly, thereby enabling us to systematically increase or decrease the volume that a participant consumes relative to the amount that he or she observes they have consumed. In so doing, a mismatch can be achieved between i) the proximal effects of the soup in the GI tract and, ii) an episodic memory that forms around the amount consumed during a meal. By exploring the interaction between actual and remembered amounts we sought to quantify their relative contribution to hunger and fullness over a three-hour period.

A second objective was to establish the extent to which our memory manipulation impacts beliefs about the soup at a subsequent test session. Recently, we and others have explored a range of phenomena relating to ‘expected satiation’ – the extent to which a food is expected to deliver fullness when compared with other foods on a calorie-for-calorie basis [27]–[32]. Expected satiation is an excellent predictor of the energy content of self-selected meals and more important than how much a food is liked [27].

Expected satiation can be viewed as an example of ‘semantic memory’ or ‘general knowledge’ about the world. By contrast, ‘episodic memory’ refers to the encoding of autobiographical information relating to a specific event that is located in time. Episodic and semantic memories appear to function in distinct yet interdependent ways. For example, expisodic memory is often ‘reconstructed’ or biased by semantic memory [33]. We reasoned that expected satiation might also be subject to bias. Specifically, we predicted that the effects of our memory manipulation might influence post-meal hunger and fullness and, in turn, that this might be rememberd and bias the expected satiation of a fixed portion of soup 24-hours later. Evidence of this kind is important, because it would suggest that ‘memory for recent eating’ has the potential to influence beliefs about a food, well beyond the immediate intermeal interval.

## Materials and Methods

### Experiment Overview

Participants were tested in a between-subjects design. On arrival, they were shown either 300 ml or 500 ml of soup. Participants then consumed either 300 ml or 500 ml. An orthogonal combination of seeing either 300 or 500 ml and then eating either 300 ml or 500 ml rendered four separate conditions. ‘Incongruous eating’ was achieved by covertly manipulating soup entering or leaving the bowl during the meal. Appetite was assessed for three hours after the meal and the expected satiation of the soup was assessed approximately 24-hours later.

### Participants

One hundred volunteers (69 female and 31 male) completed the study and produced responses to an awareness questionnaire indicating that they were unaware that the soup bowl had been modified. Six other participants reported a degree of awareness and were rejected and replaced on this basis. All were staff or students at the University of Bristol. Volunteers had a mean BMI of 23.4 (*SD* = 3.46), 22 were overweight and five were obese.

Participants were recruited by email. Vegetarians and vegans were excluded, together with anyone who declared a food allergy and/or intolerance. All received ten pounds Sterling for their assistance. The study was approved by University of Bristol Faculty of Science Human Research Ethics Committee. All provided written informed consent before assisting with the study.

### Soup and Soup-bowl Apparatus

**Figure 1:**
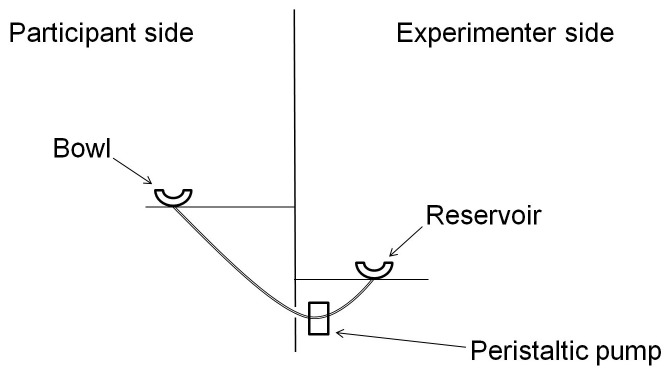
Depiction of the self-refilling soup bowl apparatus.

Soup was added or removed from a transparent soup bowl using a peristaltic pump (see Figure 1). The soup bowl was presented in front of the volunteers and it was fixed to a table. A tall screen was positioned at the back of the table. This separated the participant from both the experimenter and a second table, supporting the pump and a soup reservoir. Throughout the experiment, the volunteers were unable to see beyond the screen.

The bottom of the soup bowl was connected to a length of temperature-insulated food-grade tubing. This connection was hidden from the participants using a tablecloth. The tubing fed through a hole in the table (immediately under the bowl) and connected to the pump and then to a reservoir of soup via a hole in the screen. The experimenter was able to manipulate the direction and rate of flow using an adjustable motor controller that was attached to the pump. The pre-heated soup was ‘creamed tomato soup’ (supplied by Sainsbury’s Supermarkets Ltd., London; 38 kcal/100 g).

### Expected Satiation

Our measure of expected satiation was based on a ‘method of adjustment,’ and is described in detail elsewhere [27], [28]. Briefly, a 400-ml portion of soup was placed in front of the participant and a photograph of a ‘comparison food’ was displayed on a computer screen. The participants were instructed to match the food picture so it would ‘fill them up as much as the bowl of soup in front of them.’ Participants were instructed to taste one spoonful of soup from the bowl and then adjust the amount of comparison food on the screen. The left arrow-key (on a keyboard) caused the portion size of the comparison food to decrease. The right arrow-key caused the converse. The pictures were loaded with sufficient speed that continuous depression of the left or right arrow key gave the appearance that the change in portion size was ‘animated.’ Each trial started with a different and randomly selected portion size. Participants completed four trials, with each of four comparison foods: chicken tikka masala curry and rice (a dish that is very popular in the UK), margarita pizza, oven-baked fries, and egg penne-pasta mixed with pasta sauce. Comparison foods were presented in a counterbalanced order across participants. Portion sizes of the comparison food images ranged from 50 kcal to 1250 kcal and were spaced in equal logarithmic steps. Fifty images were taken of each food on the same white plate (255 mm diameter), with photographic conditions maintained as constant as possible.

### Procedure

Testing took place individually between 11∶00 and 14∶30 hours. Volunteers attended two sessions approximately 24 hours apart. They were asked to abstain from eating for three hours before the initial session, and to confirm that they had complied with this request on arrival. Hunger and fullness were then assessed using 100-mm visual-analogue scales labelled ‘How [hungry/full] are you right now?’ and anchored ‘not at all [hungry/full]’ to ‘extremely [hungry/full].’ Participants were also asked to report how long it had been since their last meal. Using this assessment of hunger, the participants were then pseudo-randomly assigned to one of the four conditions using a minimisation method [34], [35] with a 4∶1 element of chance. This method made it probable that the groups would be balanced for age, gender, and initial hunger.

Participants were then taken to a testing booth where a bowl of soup was waiting. They were instructed to avoid touching the bowl and to eat until the volume of soup remaining matched a line on the side of the bowl. The line ensured that eating terminated with 100 ml of soup remaining, thereby obscuring the bottom of the bowl. To accommodate for this amount, across conditions, the initial starting portion was 100 ml larger than the amount consumed. All participants were informed that eating their prescribed portion was a mandatory part of the procedure.

After the meal, hunger and fullness ratings were then taken once again. Participants were then given a pack containing an information sheet with written instructions, a food diary for the rest of the day, and three hunger and fullness rating scales, labelled one-hour, two-hours and three-hours. They were also issued a buzzer that sounded every hour for three hours. On each occasion, they were instructed to complete the appropriate hunger and fullness rating. This procedure has been used in previous studies in our laboratory and compliance with these instructions has been found to be high [36], [37]. Over this three-hour period the volunteers were instructed to abstain from eating and from drinking calorie-containing beverages.

Approximately 24 hours later the participants were shown a bowl containing 400 ml of tomato soup and evaluated its expected satiation. They then completed the Dutch Eating Behaviour Questionnaire (DEBQ) [38] which comprises three sub-scales that assess aspects of everyday dietary behaviour (dietary restraint, external eating, and emotional eating). The participants then completed a two-part questionnaire to assess demand awareness. The first section required participants to guess the purpose of the study. In the second section the participants were asked to indicate ‘yes’ or ‘no’ in response to the question ‘was the soup manipulated in any way?’ Participants who selected ‘yes’ were then asked to explain their response.

Finally, a measure of their height and weight was taken. Debriefing took place by email, after all of the data had been collected.

### Data Analysis

One-way ANOVA was used to explore evidence for differences in baseline characteristics across conditions. Specifically, we assessed BMI, age, initial hunger, and scores on the three subsections of the DEBQ.

To explore hunger ratings in the inter-meal interval, we used a mixed-model ANOVA with time (0, 60, 120, and 180 minutes) as a within-subjects factor and amount seen (300 ml or 500 ml) and amount eaten (300 ml or 500 ml) as between-subjects factors. The same approach was also used to analyse ratings of fullness. Where we found a significant main effect or interaction term we used ANOVA to scrutinise the effects of perceived and actual amounts, at each time point, separately. In all cases, to reduce error variance, we included baseline ratings as a covariate where a significant correlation existed between a dependent measure and its baseline counterpart. Finally, for each participant, we calculated an expected-satiation score (kcal) by taking an average (mean) of the selected comparison foods. Higher values indicate that the soup was expected to deliver greater satiation in the second test session. To explore the effects of amount seen and amount eaten we submitted these expected satiation scores to a 2 x 2 ANOVA.

## Results

### Participant and Baseline Characteristics

Twenty-five participants were recruited into each condition. Across conditions, we found no significant differences in BMI, initial hunger, initial fullness, age, and scores on the separate subsections of the DEBQ (all *p*>0.24). Table 1 shows related means, together with the gender distribution in each condition.

**Table 1:** Baseline and participant characteristics.

	Condition			
	see 500 ml/eat 300 ml	see 300 ml/eat 500 ml	see 500 ml/eat 500 ml	See 300 ml/eat 300 ml
Age (y)	24.2 (8.5)	25.7 (8.3)	26.9 (8.8)	27.6 (10.4)
BMI	22.4 (2.5)	23.9 (4.4)	23.8 (3.7)	23.4 (2.9)
DEBQ				
Restrained eating	2.6 (0.86)	2.6 (0.78)	2.4 (0.62)	2.6 (0.91)
External eating	3.5 (0.55)	3.2 (0.44)	3.4 (0.72)	3.5 (0.66)
Emotional eating	2.5 (0.67)	2.1 (0.62)	2.3 (0.75)	2.4 (0.75)
Initial hunger (mm)	62.7 (23.3)	58.6 (25.1)	68.4 (16.6)	67.6 (13.3)
Initial fullness (mm)	23.9 (20.4)	27.7 (19.3)	17.4 (18.9)	21.0 (13.8)
Gender (n)	F = 17/M = 8	F = 17/M = 8	F = 16/M = 9	F = 19/M = 6

Means (+/− *SD*) and frequencies (n) are shown as appropriate.

### Appetite Ratings in the Inter-meal Interval – day One

**Figure 2:**
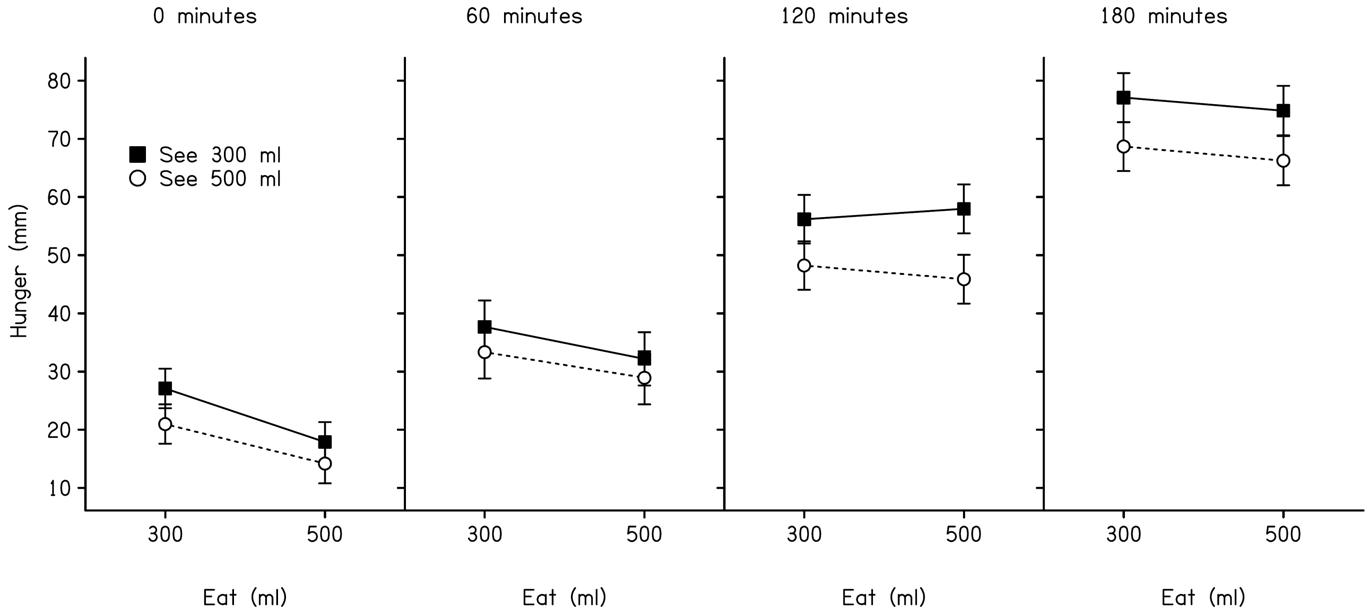
Estimated marginal means (+/− *SEM*) for hunger ratings (0–100 mm) taken 0, 60, 120, and 180 minutes after consuming the soup. Separate values are provided for participants in each condition.

Hunger increased significantly during the inter-meal interval (*F*(3,285) = 9.39, *p*<0.001). However, it increased to a lesser extent in volunteers who saw 500 ml of soup than in those who saw 300 ml (*F*(1,95) = 4.7, *p = *0.033). By contrast, the main effect of amount eaten (300 ml or 500 ml) failed to reach significance (*F*(1,95) = 1.54, *p = *0.22). Figure 2 shows estimated marginal means for hunger ratings at separate time points in post-meal interval. Separate values are provided for participants in each condition. A post-hoc analysis of ratings at each interval (0, 60, 120, and 180 minutes) exposed a significant main effect of amount eaten at 0 minutes (*F*(1,95) = 5.57, *p*<0.05), and a significant effect of amount seen at 120 and 180 minutes (*F*(1,95) = 5.78, *p*<0.05 and *F*(1,95) = 4.06, *p*<0.05, respectively). All other main effects and interaction terms failed to reach significance (all *p*>0.15). Our analysis of fullness ratings revealed a significant decrease in fullness over time (*F*(3,285) = 94.6, *p*<0.001). However, we failed to identify a main effect of either amount seen or amount eaten (*F*(1,95) = 0.36, *p* = 0.55 and *F*(1,95) = 0.65, *p* = 0.42, respectively), and the interaction between amount seen and amount eaten also failed to reach significance (*F*(1,95) = 1.83, *p = *0.18). Accordingly, no further analyses were conducted on ratings of fullness.

### Expected Satiation – day Two

**Figure 3:**
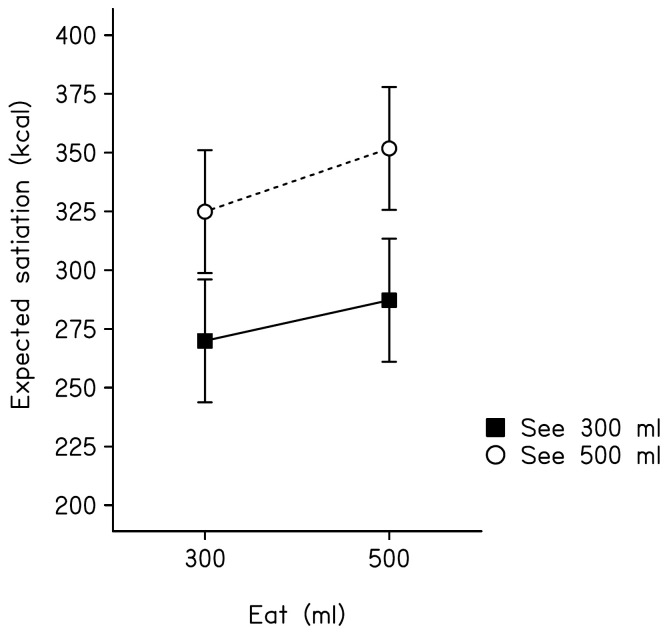
Mean (+/− *SEM*) expected satiation scores. Separate values are provided for participants in each condition.

Figure 3 shows mean (+/− *SEM*) expected satiation scores across conditions. Participants who saw 500 ml of soup in the previous test session expected it to deliver significantly more satiation than those who previously saw 300 ml of soup (*F*(1,96) = 4.95, *p*<0.05). This is the case despite the fact that all volunteers evaluated the same 400 ml portion of soup on day 2. The main effect of amount eaten and the interaction between amount eaten and amount seen both failed to reach significance (*F*(1,96) = 0.69, *p* = 0.4 and *F*(1,96) = 0.07, *p = *0.78, respectively).

### Demand Awareness

In response to the open-ended question about the purpose of the study, 53% thought that the study was assessing the extent to which soup is filling in comparison to other types of foods, 18% thought that the study was investigating the validity of ratings of hunger and fullness, and 13% suggested that the study was investigating the relationship between expectations of fullness and actual fullness. Other participants offered alternative suggestions, none of which related to the objectives of the study. Six participants failed to complete the question.

In response to the question ‘was the soup manipulated in any way?’ six participants confirmed that it had been artificially refilled or drained. As noted above, these were excluded and replaced. A further 19% responded ‘yes’ to this question. However, none of these participants commented on a change in volume (most referred to the viscosity of the soup).

### Manipulation Check

Two additional studies were conducted to demonstrate that; i) participants were able to discriminate between 300 ml and 500 ml bowls of soup and, ii) during the inter-meal interval participants had different memories of the amount of soup that they consumed. In each case we used the same equipment and materials as in the main study.

### Volume Discrimination

Twenty participants were tested. Half were presented with a bowl containing 300 ml of soup. The other half were given 500 ml. They were then instructed to imagine that they would be consuming the entire bowl of soup. The bowl was then removed and participants were presented with a pre-weighed empty bowl and a jug containing 1 litre of soup. They were asked to recall the amount of soup they had seen previously and to serve that amount into the empty bowl (participants were not pre-warned that their memory would be tested in this way). In a second task, participants were presented with a soup bowl containing either 300 ml or 500 ml, a jug of soup, and an empty bowl. They were instructed to pour soup into the empty bowl until they were equal in volume.

Independent samples *t* tests confirmed that the groups were well balanced. We found no significant differences in gender, age, hunger, or fullness (all p>0.05). Relative to participants in the 300-ml condition, those who were shown 500 ml remembered their sample as being significantly larger (*t(18)* = −2.97, p = 0.008) and they matched to a significantly larger volume (*t(11.48)* = −8.42, p<0.001). Mean (+/−SD) values are provided in Table 2.

**Table 2:** Portion size estimates (ml) from participants who saw 300 ml or 500 ml of soup.

	See 300		See 500	
	Mean	S.D.	Mean	S.D.
Portion-size memory (immediate)	433.8	99.7	572.6	109.4
Matching task	491.9	54.9	648.2	20.6

### Portion-size Memory During the Inter-meal Interval

To demonstrate that this discrimination persists in the memories of participants during the inter-meal interval, we recruited a further 20 participants and assigned them alternately to one of the two ‘incongruous eating’ conditions (either see 300 ml/eat 500 ml or see 500 ml/eat 300 ml). The protocol was identical to the first session in the main experiment except that participants were asked to return to the laboratory two hours after consuming the soup. They were then presented with a pre-weighed bowl containing 100 ml of soup (the amount remaining at the end of their meal) and a jug containing 1 litre of soup. They were then asked to recall and then serve the volume of soup they had consumed earlier. Across the two conditions, independent-samples *t* tests confirmed no significant differences in age, BMI, DEBQ restraint, DEBQ externality, DEBQ emotional eating, baseline hunger, baseline fullness, and gender (all *p*>.05). Those who saw 300 ml but consumed 500 ml remembered consuming a significantly smaller portion than those who initially saw 500 ml but consumed 300 ml of soup (*t*(18) = −3.80,*p* = 0.001). Respectively, the mean amount estimated was 475.3 ml (*S.D.* = 71.0) and 600.9 ml (*S.D.* = 76.5).

## Discussion

For the first time, we attempted to quantify the independent role of cognition (episodic memory) as a determinant of satiety in humans. This was achieved by covertly manipulating the amount of soup entering or leaving a soup bowl during a meal. Immediately after consuming the soup, hunger ratings were suppressed. Participants who consumed 500 ml reported a greater reduction in hunger than those who consumed 300 ml. We attribute this to the immediate proximal effect of the food promoting neural and endocrine signalling [39]–[41]. By contrast, at meal termination, we found little evidence that hunger was mediated by a memory for the amount of food that had been presented at the beginning of the meal (300 ml or 500 ml). This result contrasts a previous finding interpreted as marked insensitivity to the physical volume of food served from a self-filling soup bowl [26].

Further into the inter-meal interval, a different pattern of results was observed. Two and three hours after the meal, hunger was no longer predicted by the actual amount consumed. This was the case despite the fact that participants ate either 300 ml or 500 ml of soup. Instead, where differences in hunger were observed, these related to the perceived amount at the beginning of the meal. Specifically, participants who were shown 500 ml of soup experienced greater satiety than those who were shown 300 ml. This result accords with Higgs’ original proposition that satiety is influenced by memory for a recently consumed meal [13]. In particular, it fits well with a previous observation that effects of memory recall are evident three hours after eating but not after only one hour [15]. More generally, our findings are noteworthy because they reveal that the role for memory processes is substantial and that it can be exposed even without the need to explicitly cue a memory of recent eating.

The prospect that satiety is influenced by memory for recent eating is consistent with studies exploring the role of expectations around mealtime. Several studies show that beliefs about the content or energy density of a meal can have a marked effect on subsequent hunger and fullness [37], [39], [42], [43]. One suggestion is that information about a meal may trigger ‘meal schema’ that influences intake at a subsequent meal [44]. For example, Capaldi and colleagues have reported differences in subsequent intake after eating a food described as either a ‘meal’ or as a ‘snack’ [45]. Our findings also add to emerging evidence that the retrieval of food-related imagery can impact appetite and energy intake directly [46].

One possibility is that memory for recent eating serves a purpose. Specifically, it may help to interpret post-ingestive signals by attributing them to a recently consumed meal [15], [23]. We suggest that this process enables a ‘tuning’ or contextualisation of the interoceptive signals that are generated by ‘satiety hormones’ such as cholecystokinin (CCK) [47]. One of the important advantages of our approach is that it enables us to isolate and estimate the impact of post-ingestive feedback after controlling for effects of memory for recent eating. Two and three hours into the inter-meal interval, participants who consumed very different amounts of soup (300 ml or 500 ml) reported broadly similar hunger, suggesting blunted sensitivity based on post-ingestive feedback alone. Consistent with this interpretation, amnesiacs appear to experience a disconnect between feelings of hunger and appetitive behaviour [22]. This failure to recognise and integrate visceral sensations also accords with animal hippocampal-lesion studies showing an impaired ability to use interoceptive states to predict a shock or a reward in ambiguous environments [48], [49]. In particular, one suggestion is that the hippocampus is responsible for inhibiting food intake and that this process can be conceptualised as an example of negative occasion setting [9].

These ideas are important and well grounded. Nevertheless, they remain largely untested in humans. In particular, an opportunity exists to explore obese/lean differences in memory function and appetite control. One hypothesis is that diets that are high in saturated fat impair hippocampal function and that this leads to a deficit in memory performance [10]. This results in weakened inhibitory control, leading to greater consumption of high-fat foods, further impairment, and further weight gain [9]. In future, this proposition might be explored by comparing the effects of our manipulation in consumers of high- and low-fat diets and/or in patients with Alzheimer’s disease.

A potential concern is that the manipulation of perceived intake amounts to a form of deception that tells us little about normal appetite regulation. In response, we note that our volunteers were unaware that the volume of soup had been manipulated. This makes it very difficult to attribute our findings to a simple demand characteristic. An important issue relates to whether the memory for recent eating can be modified. Many everyday behaviours are supported by implicit memory [50] - in other words, memories that facilitate performance without the need for conscious or intentional recollection of those experiences (*e.g.,* walking to work). In this context, learning is often regarded as ‘incidental’ because it occurs without conscious effort. Memory for recent eating might also be influenced by explicit processes that are under conscious control. If this is the case then an opportunity exists to enhance satiety by avoiding distraction and encouraging encoding during a meal. In a recent study, Higgs and Donohoe showed that focusing on the sensory characteristics of a food (while eating) leads to a reduction in intake at a subsequent meal [51]. They do not distinguish between implicit and explicit processes. Nevertheless, this is an exciting finding and one that holds promise as the basis for a novel therapeutic intervention.

Our manipulation check indicates that participants are able to discriminate between a 300 ml and a 500 ml portion of soup and that this ability is also expressed in memory for these portions, both immediately after exposure and after a two-hour interval. This is critical, because it shows that memory is differentially influenced by our manipulation, even though participants are never instructed to encode the amount that they have consumed. In of itself, this does not demonstrate a causal relationship between hunger and memory for recent eating. However, this would seem a parsimonious explanation for our findings. Nevertheless, two alternatives merit consideration. First, the effect of perceived volume reflects subtle differences in the capacity of the (perceived) large and small portion to elicit a conditioned cephalic phase response at the time of ingestion [52], [53]. In relation to this idea, we note that the effect of perceived volume was evident only after a delay of two hours and not immediately after eating, as might be expected were this the case. Nevertheless, it is clear that ‘high level’ beliefs and cognition can influence stomach emptying and endocrine responses to foods and beverages [54] and we recommend that these measures should be included in future research. A second possibility is that perceived volume influenced mood. Specifically, participants who saw a 300-ml portion may have been disappointed by its small size and responded to this negative response by rating their hunger higher than those who saw a 500-ml portion. In relation to this proposition, again, we suggest that a ‘protest vote’ would be more likely immediately after receiving the small portion. Instead, both immediately and at one-hour post-consumption, participants experienced a similar reduction in hunger irrespective of whether they saw 300 ml or 500 ml. Notwithstanding this point, to eliminate this hypothesis with certainty we would recommend that measures of mood and ‘portion satisfaction’ should be included in future protocols.

In addition to the immediate effects of memory on post-meal hunger and fullness, we also assessed effects on the expected satiation of a fixed portion of soup (400 ml) at a subsequent test session. Regardless of amount eaten, those participants who initially saw a smaller portion of soup (day 1) then went on to expect the 400 ml portion to be relatively less satiating (day 2). A likely explanation is that participants were biased by their recent post-ingestive experience. Those who initially saw a large portion then went on to experience a greater reduction in hunger. This memory for hunger then biased estimates of expected satiation 24-hours later. Although this interpretation remains to be tested formally, it is consistent with models that characterise the retrieval of abstract knowledge (expected satiation included) in terms of multiple activation of episodic memory traces [55]. Indeed, recent models emphasis the importance of recency in this form of learning [56].

Previously, we have shown that expected satiation is dynamic and it ‘drifts’ over time [29], [31]. Shifts are more likely to involve an increase than a decrease in expectations. However, once learned, these expectations may be preserved over long periods [57], perhaps even permanently. In future, it would be interesting to measure the extent to which our manipulation leads to sustained changes in expected satiation and the extent to which this generalises to other types of soup. We also note that memories are more likely to be retrieved if retrieval takes place in the same environment in which a memory was encoded [58]. In our experiment, participants were tested in the same environment on both days. It remains to be determined whether a shift in expected satiation is dependent on this kind of context-dependent memory.

Finally, memory for recent eating is helpful because it enables us to draw on beliefs about a food, and in particular, beliefs relating to post-ingestive consequences. These expectations are likely to be governed by flavour-nutrient associations that are refined over time as we interact with individual foods [59]. It follows that any disruption to flavour-nutrient learning will promote imprecise caloric regulation (impoverished adjustment of energy intake from one meal to the next). In a series of intriguing studies, Davidson and Swithers have experimentally manipulated the extent to which sweet tastes, viscosity, and fat predict positive nutritive post-ingestive consequences [60]–[63]. Consistently, animals that are exposed to an ‘inconsistent’ diet are found to increase food intake and bodyweight. This raises important questions about our own diet and the use of fat substitutes and artificial sweeteners in many manufactured foods. The prospect that these foods disrupt our memory for recent eating warrants attention and this represents a natural extension of the work that we present here.
